# Health-Related Quality of Life in Patients with CVID Under Different Schedules of Immunoglobulin Administration: Prospective Multicenter Study

**DOI:** 10.1007/s10875-019-0592-5

**Published:** 2019-01-15

**Authors:** Federica Pulvirenti, Francesco Cinetto, Antonio Pecoraro, Maria Carrabba, Ludovica Crescenzi, Raffaella Neri, Livia Bonanni, Giovanna Fabio, Carlo Agostini, Giuseppe Spadaro, Stefano Tabolli, Albert Farrugia, Isabella Quinti, Cinzia Milito

**Affiliations:** 1 Department of Molecular Medicine, Sapienza University of Rome, Rome, Italy; 2 Department of Medicine DIMED, University of Padova, Padua, Italy; 3 Department of Translational Medical Sciences and Center for Basic and Clinical Immunology Research, University of Naples Federico II, Naples, Italy; 4 Department of Internal Medicine, Fondazione IRCCS Ca’ Granda Ospedale Maggiore Policlinico, Milan, Italy; 5 Faculty of Medicine, Dentistry and Health Sciences, The University of Western Australia, Crawley, WA Australia

**Keywords:** Health-related quality of life, common variable immunodeficiency, immunoglobulin replacement treatment, CVID_QoL, patient empowerment

## Abstract

**Objective:**

We assessed the health-related quality of life (HRQoL) in CVID adults receiving different schedules of immunoglobulin replacement therapy (IgRT) by intravenous (IVIG), subcutaneous (SCIG), and facilitated (fSCIG) preparations. For these patients, IgRT schedule was chosen after a period focused on identifying the most suitable individual option.

**Methods:**

Three hundred twenty-seven participants were enrolled in a prospective, observational, 18-month study. Participants received IgRT for at least 2 years. The first 6 months were devoted to the educational process during which the choices related to IgRT were regularly re-assessed, and the shift to alternative regimen was permitted. During the following 12 months, clinical data were prospectively collected, and only patients who did not further modify their IgRT schedule were included in the analysis of HRQoL measured by CVID_QoL, a specific instrument, and by GHQ-12, a tool to assess minor psychiatric nonpsychotic disorders.

**Results:**

Three hundred four patients were included in the analysis. CVID_QoL global score and its dimensions (emotional functioning, relational functioning, gastrointestinal symptoms) were similar in IVIG, SCIG, and fSCIG recipients. Patients receiving IgRT by different routes of administration reported similar capacity to make long-term plans, discomfort due to therapy, and concern to run out of medications. Multivariate analysis revealed the GHQ-12 status, but not the IgRT mode of administration, as the major factor impacting on treatment-related QoL items, and a significant impact of age on discomfort related to IgRT.

**Conclusions:**

IgRT schedules do not impact the HRQoL in CVID if the treatment is established after an extensive educational period focused on individualizing the best therapeutic regimen.

**Electronic supplementary material:**

The online version of this article (10.1007/s10875-019-0592-5) contains supplementary material, which is available to authorized users.

## Introduction

Common variable immune deficiencies (CVID) are a group of diseases whose complexity in clinical presentation and treatment poses difficulties in management [1]. Significant progress has been made over the past few decades in awareness, early diagnosis, and therapeutic options, including immunoglobulin replacement therapy (IgRT), which have led to a profound change in the approach to affected patients [2, 3]. The advent of intravenously administered polyvalent immunoglobulin preparations (IVIG) in the 1980s dramatically decreased morbidity and mortality and replacement therapy is universally considered to be life-saving. Subsequently, the introduction of further therapeutic options, including the subcutaneous route of Ig administration (SCIG), which may also be facilitated by human recombinant hyaluronidase (fSCIG), and the availability of formulations containing immunoglobulin at different concentrations have led to changes in the schedule of administration [4]. In addition, the increased clinical demand for the products’ range of indications has fueled an increased requirement for plasma fractionation, leading to an expansion of the plasma donor population and has enhanced methods to improve IgG recovery [5]. Since CVID patients require therapy for life, the acceptability of the different schedules and setting for Ig administration are now considered important instruments to achieve adherence to treatment and in increasing health-related quality of life (HRQoL) in these patients [6]. Studies indicate a heterogeneity in patients’ attitudes to treatment, in particular to the role of IgRT. While a cohort of European patients with markedly different levels of access to IgRT showed similar HRQoL [6], a similar US-based assessment indicated that a regular access to therapy is beneficial in perceived health [7]. We have previously shown that such an HRQoL elicitation did not reveal any influence of Ig administration schedule in an Italian cohort, using generic and disease-specific instruments [8–10]. As with other chronic conditions, treatment of patients living with CVID could impose demands on daily life to plan and self-care management, as a result of the need to comply with complex therapeutic schedules, while balancing family or job commitments. This can result in a “burden of treatment” [11, 12]. Hence, the importance of defining a tailored immunoglobulin treatment plan for each patient’s situation has been shown [13–15], with clear implications on patient well-being [16, 17]. Our own work indicates that patients with CVID manifest substantial restrictions and poor HRQoL, which worsen over the time, mainly due to CVID-associated clinical conditions [8–10].

In order to identify problems related to IgRT route of administration, which may impact on the HRQoL of patients, we designed and carried out a prospective observational study on the HRQoL of adult patients with CVID. This investigation followed a long period of patients’ education and training aimed to establish the best treatment option. HRQoL was assessed by a recently validated new instrument: the CVID_QoL questionnaires [10], together with the GHQ-12, a tool to detect nonpsychotic, minor psychiatric disorders, such as depression and anxiety [18]. The study sought to generate information to help health care professionals to understand factors that may impact on patient’s everyday life and possibly contribute to maximizing patient empowerment and satisfaction with care, while minimizing the impact of illness.

## Methods

### Population Analyzed

Eligible patients were adults aged > 18 years with a diagnosis of CVID (http://esid.org/Working-Parties/Registry/Diagnosis-criteria) under IgRT for at least 2 years before enrollment. All patients were regularly followed by university hospital care centers for adult primary immune deficiencies in Rome, Naples, Padua, and Milan. Exclusion criteria included the following: inability or unwillingness to provide written informed consent and refusal to complete the HRQoL questionnaires. The Ethical Board of the Sapienza, University of Rome approved this study. The study was performed in accordance with the Good Clinical Practice guidelines, the International Conference on Harmonization guidelines, and the most recent version of the Declaration of Helsinki.

### Study Design

**Fig. 1:**
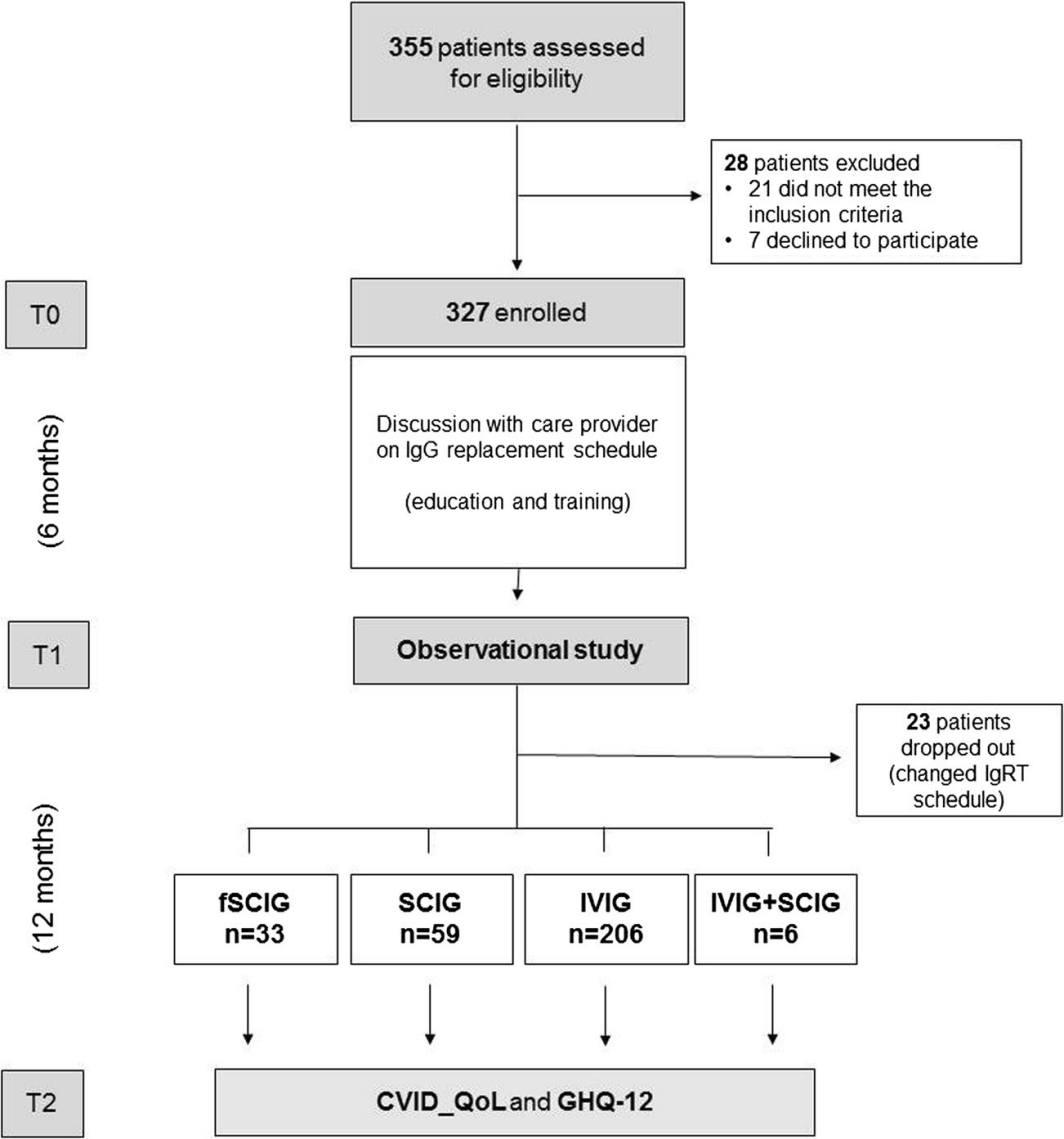
Study design flow-chart. Abbreviation: CVID_QoL, common variable immunodeficiency quality of life questionnaire; fSCIG, facilitated subcutaneous immunoglobulin; GHQ-12 GHQ-12, questionnaire; IVIG, intravenously-administered immunoglobulins; IVIG + SCIG, immunoglobulin replacement therapy by combined intravenous and subcutaneous route of administration; IgRT, immunoglobulin replacement treatment; SCIG, subcutaneous Immunoglobulins

Prospective, observational, multicenter, 18-month study (Flow Chart, Fig. 1). The study was designed to address the impact of the route of IgRT administration on CVID HRQoL. To assess this need, all CVID patients attending the participating care centers and fulfilling the inclusion criteria were invited to participate in the study. At enrollment (T0), patients signed the written informed consent. Afterward, the first 6 months of the study (“equilibration”) was devoted to the education and training of patients. The aim of the education process was to discuss the possible choices related to IgRT administration, including setting, route, intervals, possible adverse reactions, and participant lifestyle pattern. This process was intended to optimize the IgRT treatment program according to the participants’ individual needs, before assessing the HRQoL. During the “equilibration,” physicians performed at least three clinical visits to re-evaluate and possibly to modify the choice of IgRT based on individual preferences, tolerability, and acceptability. All patients continued to be monitored for their clinical status according to the Italian guidelines [19]. Patients who changed their immunoglobulin administration regimen were trained by expert nurses. Patients who shifted to fSCIG/SCIG (or their caregivers) self-administered Ig under nurse supervision until their ability was considered satisfactory both from themselves and nurses (at least three settings). At the end of the 6-month period (T1), patients were included in a 12-month observational study on their HRQoL. At T1, a set of variables was recorded for each patient including: gender, date of birth, date of CVID diagnosis, Ig serum levels at diagnosis, and IgG trough levels (TL). At the end of the observational period (T2), patients completed the CVID_QoL questionnaire, a disease-specific tool recently validated to assess HRQoL in CVID [10]. On the same day, patients completed the GHQ-12 questionnaire, a screening device for the identification of minor psychiatric disorders, such as anxiety and depression [18]. During the observational period, participants visited to the clinics every 3 months, and no medical procedures were performed outside those provided for individual care management. Patients who changed their IgRT schedule during T1–T2 were excluded by the analysis. At the end of the observational period (T2), clinical, immunological, and treatment data related to the period T1–T2 were collected from the medical records, including adherence. Adherence was defined as being able to maintain the established dose and interval of IgRT for more than 90% of administrations.

### Questionnaires

The CVID_QoL is a self-administered questionnaire developed and validated in Italy in 2016 and translated into US English [10]. Study on translation and cross-cultural validation are currently ongoing in several countries. The instrument includes 32 items in a Likert-type or forced-choice format and measures health on three multi-item dimensions: emotional functioning (EF), relational functioning (RF), and gastrointestinal and skin symptoms (GSS). It includes also a summary measure named global CVID_QoL. The EF dimension includes 19 items on patient feelings, on difficulties related to CVID manifestations, on Ig replacement treatment and relationships with other patients, and on fears concerning evolution of disease or adverse reaction to treatments. The RF dimension includes nine items on patients’ relationship with relatives and not-affected people; gastrointestinal and skin symptom (GSS) domain includes four items on complications related to gastrointestinal manifestation or skin disease. The content of the CVID_QoL questionnaire is summarized in Supplementary Table 1. Response options are formulated using a 5-point scale, with 0 = “never” and 4 = “always,” with higher values indicating increasing disability. The CVID_QoL global score and scores for each dimension are defined as the sum of all scores of each item transformed as a percentage of the maximum possible score. Additionally, to better evaluate the impact of IgRT on HRQoL, we analyzed the score of single questions (Q) possibly related to IgRT: Q.5; difficulties to make long-term plans; Q.9: discomfort/pain on joints; Q.11: being afraid to run out of medications; Q.12: being concerned on adverse events to Ig treatment; Q.15: feeling less independent than usual; Q.24: being bothered by Ig treatment; Q31: being troubled by relationships with other CVID patients; Q32: feeling tired.

The GHQ-12 [18] is a self-administered, 12-items questionnaire, designed to measure psychological distress and to detect current nonpsychotic, psychiatric disorders, such as depression and anxiety. Answers are given on a 4-point scale. When scored with the binary method (0–0–1–1), the GHQ-12 can be used as a screening tool yielding final scores that range from 0 to 12. Operationally, patients scoring 4 or more were considered as “GHQ-positive (GHQ+)”/at risk of anxiety and depression.

### Statistical Analysis

Demographics of the CVID database are summarized with descriptive statistics. Sociodemographic, immunological, and clinical variables were compared between patients receiving different immunoglobulin treatments. For repeated measures (i.e., IgG through level), the individual mean value was calculated. We first conducted a univariate analysis to assess the impact of variable of interest related to the eight questions of CVID_QoL on IgRT. The *χ*^2^ test was used for categorical variables, and the *t* test was used for continuous variables. A multiple linear regression model was used to simultaneously evaluate the impact of selected variables on the eight questions of CVID_QoL related to IgRT. The selection of these variables was based on the underlying conceptual framework than on pure statistical significance and included age, sex, route of IgRT administration, GHQ status, COPD, enteropathy, occupation, and education level. The standardized beta coefficient was used to compare the magnitude of association. The statistical significance was set at the conventional level of *p* < 0.01 to reduce the chance of false positives. Statistical Package for Social Sciences version 15 (SPSS Inc., 233 South Wacker Drive, 11th Floor, Chicago) was used for the analysis.

## Results

### Patient Population and Immunoglobulin Administration

As shown in Fig. 1, 355 CVID patients were assessed for eligibility and 28 were excluded (21 for not meeting inclusion criteria and seven for refusal to participate). Three-hundred twenty-seven participants were enrolled in the educational/training 6 months. Forty-five subjects decided together with their doctor to change the mode of administration during the education period: 29% participants shifted from IVIG to SCIG, 27% from IVIG to fSCIG, 20% from fSCIG to IVIG, 11% from SCIG to fSCIG, and 13% from SCIG to IVIG. In the following 12 months (observational study), 23 patients decided to further modify the IgRT schedule defined and then they were excluded from the analysis (reasons for changing are listed in Supplementary Table 2).

Three-hundred and four CVID patients (mean age = 47 years, range = 18–82, 1:1 male to female ratio) concluded the 12-month observational period and completed the CVID_QoL and the GHQ-12 questionnaires. The largest group of participants included patients under IVIG (206 subjects; 67.7%), followed by patients receiving SCIG (59 subjects; 19.4%) and fSCIG (33 subjects; 10.0%). Six patients (1.9%) received replacement therapy by SCIG plus IVIG (combined IgRT), because they were unable in the past to maintain an acceptable IgG trough level (> 500 mg/dL) by other schedules of administration (Fig. 1).

At T1, groups under fSCIG, SCIG, and IVIG were comparable for IgG trough levels and for length of CVID disease. Patients receiving IVIG were older than those receiving fSCIG (49.2 ± 16.1 years vs 40.3 ± 11.2 years, *d* = 0.64, *p* = 0.006). Compared to patients under fSCIG and SCIG, patients receiving IVIG had lower IgG serum levels at diagnosis, IgA serum levels, and frequencies of switched memory B cells (CD19^+^CD27^+^IgM^−^IgD^−^), even if these differences were not statistically significant (Table 1).

**Table 1:** Characteristics of CVID patients by type of immunoglobulin treatment at T1

	fSCIG *n* = 33	SCIG *n* = 59	IVIG *n* = 206	SCIG + IVIG *n* = 6
Age (years), mean (SD)	40.3 (11.2)^c^	45.2 (13.1)	49.2 (16.1)^a^	39.2 (13.1)
Sex (females), *n* (%)	11.5(33.1)	26.1 (44.2)	113.2 (55.2)	4.5 (67.2)
Occupation (employed), *n* (%)	25 (75)	34 (57)	99 (48)	3 (50)
Education (≥ high school), *n* (%)	30 (90)	48 (81)	150 (73)	5 (83)
Time (years) from PID diagnosis, mean (SD)	11.2 (9.2)	9.0 (7.1)^d^	12.3 (11.1)	19.1 (11.0)^b^
Ig serum levels at diagnosis:				
- IgG (mg/dL), mean (SD)	326.3 (183.5)	313.3 (161.2)	246.5 (179.2)	160.2 (186.3)
- IgM (mg/dL), mean (SD)	23.2 (21.2)	27.2 (24.6)	26.1 (38.6)	5.2 (5.1)
- IgA (mg/dL), mean (SD)	39.1 (52.3)	28.2 (33.5)	22.3 (58.3)	18.2 (26.2)
IgG trough serum levels (mg/dL), mean (SD)	713.3 (115.1)	745.0 (109.2)^d^	725.1 (180.2)	600.5 (122.3)^b^
CD3+ CD4+/mm3, mean (SD)	785.3 (542.1)	740 (381)	624.1 (343.2)	935.7 (763.1)
CD19+ (%), mean (SD)	8.6 (8.3)	12 (17)	13.1 (23.2)	6.1 (1.0)
CD19+ CD27+ IgM^−^ IgD^−^ (%), mean (SD)	7.8 (14.7)	7 (10)	6.1 (14.2)	2.1 (3.2)

^a^*p* < 0.01 in comparison to fSCIG group

^b^*p* < 0.01 in comparison to SCIG group

^c^*p* < 0.01 in comparison to IVIG group

^d^*p* < 0.01 in comparison to IVIG + SCIG group

At T2, groups under fSCIG, SCIG, and IVIG were comparable for the monthly cumulative dose of immunoglobulins administered, IgG trough levels, and for antibiotic prophylaxis usage (Table 2). As expected, the average number of monthly IgG administrations was different between groups (SCIG vs fSCIG *d* = 1.72, *p* < 0.001, SCIG vs IVIG *d* = 1.81, *p* < 0.001). There was no difference in the adherence to treatment between patients receiving fSCIG (94%), SCIG (92%), and IVIG (94%). Reasons of poor adherence were all related to difficulties to maintain the established interval between administrations: fSCIG (2/33), SCIG (5/59), IVIG (12/206), and combined routes of Ig treatment (1/6).

**Table 2:** Data of CVID patients at T2

	fSCIG *n* = 33	SCIG *n* = 59	IVIG *n* = 206	SCIG + IVIG *n* = 6
IgG trough serum levels (mg/dL), mean (SD)	723.5 (143.1)	797 (159)	756.2 (214.2)	607.3 (162.1)
IgM serum levels (mg/dL), mean (SD)	28.6 (25.2)	23 (26)	30.1 (51.2)	10.5 (9.5)
IgA serum levels (mg/dL), mean (SD)	40.2 (58.2)	30 (45)	24.1 (63.2)	12.3 (24.4)
Cumulative monthly Ig dose (mg/kg), mean (SD)	317.8 (120.3)^d^	359.0 (152.5)	351.5 (148.6)	490.2 (238.1)^a^
Number of monthly administrations mean (SD)	2.1 (1.0)^b^	4.1 (1.3)^a,c^	2.0 (1.0)^b^	4.2 (2.5)
Antibiotic prophylaxis, *n* (%)	4 (12.1)	9 (15.2)	23 (11.5)	2 (33.2)
Adherent to treatment, *n* (%)	31 (93.9)	54 (91.5)	194 (94.4)	5 (83.3)

^a^*p* < 0.01 in comparison to fSCIG group

^b^*p* < 0.01 in comparison to SCIG group

^c^*p* < 0.01 in comparison to IVIG group

^d^*p* < 0.01 in comparison to IVIG + SCIG group

### CVID-Associated Morbidities

**Fig. 2:**
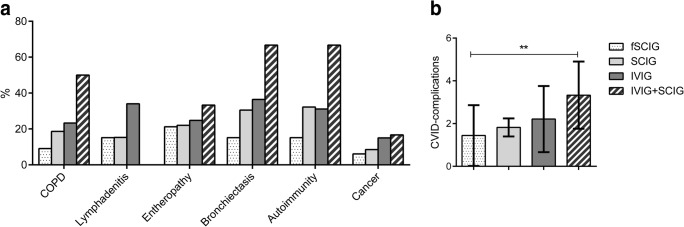
CVID-related clinical conditions (panel **a**) and mean number of co-morbidities (panel **b**) observed in CVID grouped according to their IgRT during the observational time. *p* values by the *t* test. **p ≤ 0.01. Abbreviation: CVID, common variable immunodeficiency; COPD, chronic obstructive pulmonary diseases; IVIG, intravenously-administered immunoglobulins; SCIG, subcutaneous Immunoglobulins; fSCIG, facilitated subcutaneous immunoglobulin; IVIG + SCIG, immunoglobulin replacement therapy by combined intravenous and subcutaneous route of administration

Figure 2 shows data on CVID-associated clinical conditions in the 304 patients, grouped according to the route of Ig replacement. Patients on fSCIG had the lowest frequency of associated conditions. However, significant differences were not found between groups (Fig. 2a). The group under combined therapy displayed a more severe phenotype (high rate of chronic obstructive pulmonary disease (COPD), bronchiectasis, and autoimmune diseases) and the highest number of CVID-complications (3.33 ± 1.50, *p* = 0.006, Fig. 2b).

Table 3 shows the rate of infections and the number of hospitalizations recorded among participants during the 12 months of observational period. No difference was found among groups in the number of cumulative episodes of diarrhea, acute sinusitis, bronchitis, otitis, and pneumonia. No difference was further found in the number of hospitalizations for any cause among groups. A further analysis was carried out within patients who were treated with IVIG preparations containing 5 (5%) or 10 (10%) grams/dL. Groups under IVIG 5 and 10% were comparable by demographics, IgG cumulative monthly dosage, and CVID-associated conditions. No difference in the number of infectious episodes was observed between patients under the two-treatment concentration (Supplementary Table 3).

**Table 3:** Number of acute infectious episodes and hospital admission

	FSCIG *n* = 33	SCIG *n* = 59	IVIG *n* = 206	SCIG + IVIG *n* = 6
Diarrhea, episodes-year, mean (SD)	4.1 (2.7)	3.2 (3.1)	3.3 (2.8)	4.7 (3.9)
Sinusitis, episodes-year, mean (SD)	1.6 (1.6)	1.6 (1.6)	1.7 (1.6)	2.7 (1.8)
Bronchitis, episodes-year, mean (SD)	1.3 (1.5)	1.4 (1.5)	1.3 (1.5)	1.7 (1.6)
Otitis, episodes-year, mean (SD)	0.3 (0.8)	0.3 (0.8)	0.4 (1.0)	0.3 (0.8)
Patients with pneumonia, *n* (%)	6 (18.8)	5 (8.9)	30 (14.9)	1 (16.7)
All infections requiring treatment episodes-year, mean (SD)	4.4 (5.2)^§^	3.9 (3.8)	3.8 (4.0)	9.0 (6.1)^§^
Hospital admission (all causes), *n* (%)	2 (6.1)	7 (11.9)	15 (7.3)	1 (16.7)

^§^*p* < 0.01, comparison between fSCIG group and IVIG + SCIG group

### Quality of Life

**Fig. 3:**
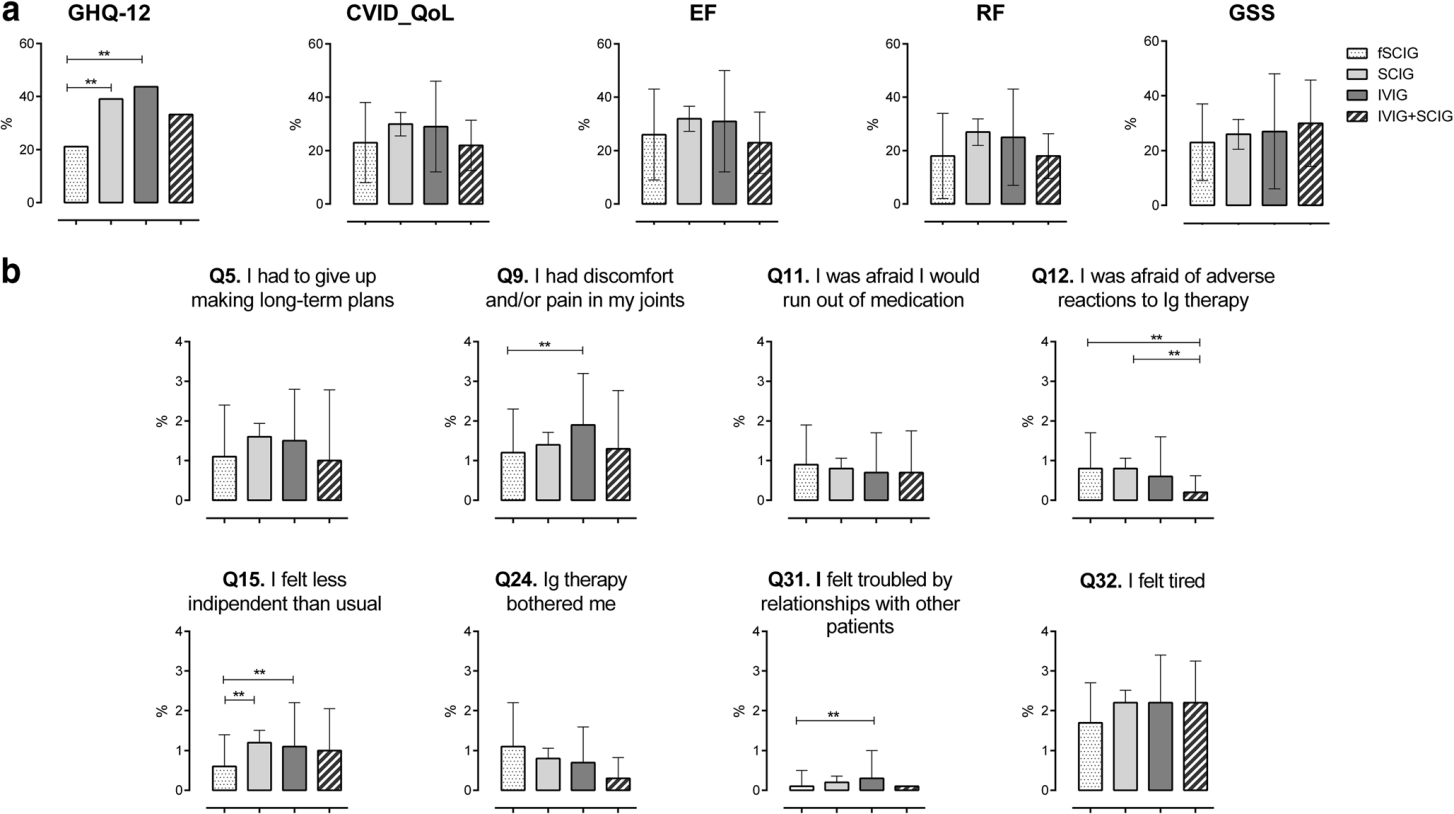
GHQ status and CVID_QoL score. Proportion of GHQ-12-positive participants and global. EF, RF, and GSS scores (panel **a**) recorded for CVID grouped according to the route of IgG replacement treatment. CVID_QoL global. EF, RF, and GSS are expressed as percentage of the respective total score with higher values indicating increasing disability. Scores of eight single items of the CVID_QoL related to the immunoglobulin treatment (panel **b**). *p* values by the *t* test. **p ≤ 0.01. Abbreviation: CVID_QoL, common variable immunodeficiency quality of life questionnaire; EF, emotional functioning; IVIG, intravenously administered immunoglobulins; fSCIG, facilitated subcutaneous immunoglobulin; GHQ-12, GHQ-12 questionnaire; GSS, gastrointestinal and skin symptoms; IgRT, immunoglobulin replacement treatment; IVIG + SCIG, immunoglobulin replacement therapy by combined intravenous and subcutaneous route of administration; Q, question; RF, relational functioning; SCIG, subcutaneous immunoglobulins

HRQoL was evaluated by the generic GHQ-12 questionnaire [18] and by the disease-specific CVID_QoL questionnaire [10] at the end of the observational period. Nearly half (46%) of participants had a GHQ-positive status (Fig. 3a). Patients under fSCIG less likely had a GHQ-positive status in comparison to patients receiving IVIG (*p* = 0.01; OR 3.2, 95% CI 1.3–7.7) and SCIG (*p* = 0.01; OR 3.0, 95% CI 1.1 to 8.4). Moreover, in the IVIG group, no difference was recorded between patients receiving 5 or 10% preparations (Supplementary Table 3). No significant differences were found in CVID_QoL global scores and in its dimensions EF, RF, and GSS between groups under different Ig route of administration (Fig. 3a). The univariate analysis of single questions related to immunoglobulins treatment (Fig. 3b) showed no difference between patients belonging to the fSCIG, SCIG, and IVIG groups in terms of difficulties to make long-term plans (Q.5), being afraid to run out of medications (Q.11), being concerned on adverse events to Ig treatment (Q.12), nor being bothered by Ig treatment (Q.24). Participants under fSCIG reported to feel “less independent than usual less frequently” (Q.15) in comparison to those under SCIG (*d* = 0.55, *p* = 0.01) or under IVIG (*d* = 0.55, *p* = 0.006). Moreover, CVID under IVIG reported significantly higher score (greater difficulties) on discomfort/pain on joints (Q.9, *d* = 0.60, *p* = 0.002) and on being troubled by relationships with other CVID patients (Q.31, *d* = 0.28, *p* = 0.004) in comparison to patients receiving fSCIG. Notably, CVID receiving both SCIG and IVIG (combined) reported a lower score related to their concern for adverse events due to replacement therapy (Q.12) in comparison to fSCIG (*d* = 0.75, *p* = 0.01) and SCIG group (*d* = 0.93, *p* = 0.01). Univariate analysis revealed the GHQ-positive status as strongly (*p* < 0.0001) linked to all IgRT-related items, except for Q.11 (“to be afraid to run out of medications,” Table 4). “Difficulties to make long-term plans” (Q.5) were associated to female gender, COPD, low educational level, and unemployed status; similarly, older patients (≥ 40 years), females, with COPD, and unemployed reported “to feel more frequently less independent than usual” (Q.15). Higher score on “discomfort/pain on joints” were recorded in older, less educated, unemployed participants and females. Older patients were more likely to be “troubled by relationships with other CVID patients.” Females, unemployed, and COPD patients reported to “feel more frequently tired” (Q.32) (Table 4). Multivariate analysis confirmed the lack of impact of the route of IgRT administration and revealed a major role of GHQ-positive status on all the eight items. The analysis also revealed a significant impact of age on “discomfort/pain on joints” (Q.9) and on being “bothered by immunoglobulins” (Q.24) (Table 5).

**Table 4:** CVID_QoL scores of selected items by sociodemographic and clinical variables

		Q.5 I had to give up making long-term plans	Q.9 I had discomfort and/or pain in my joints	Q.11 I was afraid I would run out of medication	Q.12 I was afraid of adverse reaction to Ig therapy	Q.15 I felt less independent than usual	Q.24 Ig therapy bothered me	Q.31 I felt troubled by relationship with other patients	Q32 I felt tired
Age	*p* value	0.314	< 0.0001	0.191	0.618	0.011	0.120	0.003	0.242
≥ 40 year	Mean (SD)	1.5 (1.4)	1.9 (1.3)	0.7 (1.0)	0.7 (0.9)	1.1 (1.1)	0.7 (0.9)	0.4 (0.8)	2.2 (1.2)
< 40 year	Mean (SD)	1.4 (1.2)	1.3 (1.2)	0.6 (0.9)	0.6 (0.9)	0.8 (1.0)	0.9 (1.0)	0.1 (0.4)	2.0 (1.2)
Sex	*p* value	0.001	0.009	0.881	0.449	0.007	0.221	0.112	< 0.0001
Female	Mean (SD)	1.7 (1.3)	1.9 (1.3)	0.7 (1.0)	0.7 (0.9)	1.2 (1.1)	0.8 (1.0)	0.4 (0.8)	2.4 (1.2)
Male	Mean (SD)	1.2 (1.3)	1.5 (1.2)	0.7 (1.0)	0.6 (1.0)	0.9 (1.0)	0.7 (0.9)	0.2 (0.5)	1.8 (1.2)
COPD	*p* value	0.011	0.066	0.056	1.000	0.013	0.704	0.216	0.004
Yes	Mean (SD)	1.9 (1.3)	2.0 (1.3)	0.9 (1.2)	0.7 (1.0)	1.4 (1.2)	0.8 (1.0)	0.4 (0.7)	2.5 (1.1)
No	Mean (SD)	1.4 (1.3)	1.7 (1.3)	0.7 (0.9)	0.7 (0.9)	0.9 (1.0)	0.8 (0.9)	0.3 (0.7)	2.0 (1.2)
Enteropathy	*p* value	0.046	0.140	0.512	0.289	0.106	0.408	0.986	0.128
Yes	Mean (SD)	1.8 (1.3)	1.9 (1.0)	0.6 (0.9)	0.8 (1.1)	1.2 (1.2)	0.8 (1.0)	0.3 (0.7)	2.3 (1.2)
No	Mean (SD)	1.4 (1.3)	1.7 (1.4)	0.7 (1.0)	0.6 (0.9)	1.0 (1.0)	0.7 (0.9)	0.3 (0.7)	2.1 (1.2)
Autoimmunity	*p* value	0.173	0.080	0.615	0.747	0.523	0.079	0.586	0.123
Yes	Mean (SD)	1.6 (1.3)	1.9 (1.2)	0.6 (1.0)	0.6 (0.9)	1.1 (1.1)	0.9 (1.0)	0.3 (0.6)	2.3 (1.1)
No	Mean (SD)	1.4 (1.3)	1.7 (1.3)	0.7 (1.0)	0.7 (0.9)	1.0 (1.1)	0.7 (0.9)	0.3 (0.7)	2.1 (1.2)
Education	*p* value	0.013	< 0.0001	0.131	0.103	0.078	0.382	0.197	0.257
≥ High school	Mean (SD)	1.4 (1.3)	1.6 (1.3)	0.7 (0.9)	0.6 (0.9)	1.0 (1.1)	0.7 (1.0)	0.3 (0.6)	2.1 (1.2)
< High school	Mean (SD)	1.8 (1.4)	2.2 (1.2)	0.9 (1.2)	0.8 (0.9)	1.2 (1.2)	0.9 (0.9)	0.4 (0.8)	2.3 (1.3)
Occupation	*p* value	0.002	0.001	0.401	0.048	0.001	0.819	0.307	0.013
Employed	Mean (SD)	1.2 (1.3)	1.5 (1.3)	0.7 (1.0)	0.6 (0.8)	0.8 (1.0)	0.8 (1.0)	0.3 (0.6)	2.0 (1.2)
Unemployed	Mean (SD)	1.7 (1.4)	2.0 (1.3)	0.8 (1.0)	0.8 (1.0)	1.3 (1.1)	0.8 (0.9)	0.3 (0.8)	2.3 (1.2)
GHQ status	*p* value	< 0.0001	< 0.0001	0.126	0.002	< 0.0001	< 0.0001	0.002	< 0.0001
Positive	Mean (SD)	1.9 (1.3)	2.1 (1.3)	0.8 (1.0)	0.9 (1.0)	1.4 (1.1)	1.0 (1.0)	0.5 (0.9)	2.7 (1.0)
Negative	Mean (SD)	1.0 (1.2)	1.4 (1.2)	0.6 (0.9)	0.5 (0.8)	0.6 (0.9)	0.6 (0.8)	0.2 (0.5)	1.7 (1.1)

Abbreviation: COPD, chronic obstructive pulmonary disease; GHQ, General Health Questionnaire; SD, standard deviation; Q, question (number)

**Table 5:** Impact of patients’ characteristics on CVID_QoL items related to immunoglobulins treatment by multiple linear regression

	Standardized beta	*p* value
Q5. I had to give up making long-term plans		
Route of IgRT	− 0.082	0.202
GHQ positive status	0.297	< 0.0001
Age	0.033	0.613
Sex	0.101	0.121
COPD	0.068	0.276
Enteropathy	0.111	0.075
Education	− 0.103	0.132
Occupation	− 0.063	0.366
Q9. I had discomfort and/or pain in my joints		
Route of IgRT	0.075	0.241
GHQ positive status	0.196	0.003
Age	0.189	0.005
Sex	0.045	0.492
COPD	0.022	0.729
Enteropathy	0.050	0.421
Education	− 0.115	0.094
Occupation	− 0.072	0.308
Q11. I was afraid I would run out of medication		
Route of IgRT	− 0.073	0.289
GHQ positive status	0.16	0.022
Age	0.075	0.289
Sex	− 0.032	0.649
COPD	0.135	0.045
Enteropathy	− 0.036	0.591
Education	0.001	0.994
Occupation	− 0.034	0.653
Q12. I was afraid of adverse reaction to Ig therapy		
Route of IgRT	− 0.107	0.115
GHQ positive status	0.204	0.004
Age	0.012	0.861
Sex	− 0.044	0.528
COPD	0.007	0.922
Enteropathy	0.038	0.568
Education	− 0.133	0.067
Occupation	− 0.051	0.494
Q15. I felt less independent than usual		
Route of IgRT	− 0.042	0.504
GHQ positive status	0.329	< 0.0001
Age	0.134	0.042
Sex	0.065	0.314
COPD	0.074	0.237
Enteropathy	0.059	0.34
Education	0.018	0.789
Occupation	− 0.077	0.272
Q24. Ig therapy bothered me		
Route of IgRT	− 0.13	0.047
GHQ positive status	0.301	< 0.0001
Age	− 0.191	0.005
Sex	0.042	0.529
COPD	0.024	0.708
Enteropathy	0.063	0.325
Education	− 0.108	0.121
Occupation	− 0.044	0.534
Q31. I felt troubled by relationship with other patients		
Route of IgRT	0.045	0.506
GHQ positive status	0.176	0.012
Age	0.07	0.320
Sex	0.033	0.634
COPD	0.02	0.762
Enteropathy	0.005	0.942
Education	− 0.085	0.243
Occupation	0.022	0.774
Q32. I felt tired		
Route of IgRT	− 0.034	0.583
GHQ positive status	0.412	0.0001
Age	− 0.011	0.868
Sex	0.095	0.134
COPD	0.124	0.042
Enteropathy	− 0.005	0.939
Education	− 0.025	0.702
Occupation	− 0.027	0.693

Abbreviation: COPD, chronic obstructive pulmonary disease; GHQ, General Health Questionnaire; IgRT, immunoglobulin replacement treatment; Q, question (number)

## Discussion

This observational study on adult CVID patients receiving IgRT in a real-life setting indicates that the route of Ig administration does not impact on the HRQoL if the treatment choice has been shared by patients and their physicians. Here, the best IgRT option has been established through a 6-month educative process, during which the participants’ lifestyle pattern, attitude, habits, and Ig tolerability were reviewed and discussed. This procedure was aimed at identifying new needs/product tolerability, verifying the initial choice of IgRT, and guiding towards compliance and a good adherence to treatment.

In our setting, recipients of distinct IgRT routes had similar scores in all the CVID_QoL domains, including the summary measure and its three dimensions. Participants receiving IVIG, SCIG, and fSCIG further reported similar capacity to make long-term plans, similar distress due to therapy, and similar concern to run out of medications and to experience adverse events. CVID patients receiving IVIG more frequently reported “discomfort/pain on joints” and to “feel less independent,” conditions that appeared to be mainly related to underlying anxiety and/or depression and to the older age, as revealed by multivariate analysis. Moreover, no differences in efficacy, tolerability, and HRQoL scores were found between patients receiving intravenous immunoglobulins at different concentrations. This lack of impact of the IgRT route on HRQoL in adult CVID further emphasizes the need of empowering patients in the management of their life-long disease and to focus on their psychological status [17].

The use of HRQoL tools helps to address issues related to the influence of disease and therapy from the patients’ perspective, following the principle of the patient-centered health care system [20]. Using HRQoL generic instruments in CVID, our group and others have shown that patients with CVID experience significantly lower general health and increased physical and social activity limitation [6, 8, 21–23] and that HRQoL outcome measures could help to evaluate disease progression over time and could predict morbidity and mortality in subjects with CVID [9].

The use of disease-specific tools is desirable to provide a more accurate picture of the burden of disease [10, 24, 25]. The CVID_QoL questionnaire is the first specific instrument for CVID [10], and in addition to the burden of disease due to the complex clinical picture [26], the questionnaire analyzes also the experience of the so called “burden of treatment” [11, 27]. In fact, the burden of treatment has also an impact on the needs of everyday life, in planning and managing one’s self-care, as well as complex treatments and their clinical monitoring, in addition to family/work commitments [28].

The current wide range of Ig presentations, including IVIG, SCIG, and fSCIG, should lend itself to an enhanced capacity to tailor treatment to individual patient features and preferences [14, 15]. This should improve outcomes in the treatment of CVID with Ig, given its life-long administration, as several factors may impact on replacement therapies. These include the route and setting of administration, as assessed in our study [16, 17]. The groups under different IgRT were homogeneous in terms of frequency of CVID-associated conditions and, as already shown [29], no difference was observed on the IgRT efficacy in terms of severe and mild infectious episodes and hospital admissions among different Ig formulations. It has been suggested that in case of equivalent safety and efficacy of different treatments, clinicians should take in account patient’s preference, to ensure optimal treatment adherence and ultimately get better patient’s satisfaction [30]. In addition, other factors might influence the choice of treatment, including recipients’ past experience, perceived current disease status, and therapy administration-related factors [31]. In our setting, we recorded that younger and GHQ-negative patients preferred fSCIG, whereas older and GHQ-positive patients, with a longer clinical history, preferred IVIG. This was possibly since in Italy IVIG is administered entirely in a hospital setting, allowing to a more frequent contact between patients and care givers. Young patients without problems of anxiety/depression more easily accepted the home setting and preferred the hyaluronidase-facilitated administration that allows a low number of monthly administrations [32]. In this study, we also included a group of “difficult patients” requiring both IVIG and SCIG replacement. These patients displayed a severe phenotype, with a high rate of CVID-related complications, such as COPD, bronchiectasis, and enteropathy. For these patients, Ig treatment modality mainly reflected a medical decision based on their poor clinical status and on difficulties in maintaining IgG trough levels by other schedules of administration. It is difficult to speculate on this group, because of their small number. However, despite of higher rate of infections and the combined route of administration, these patients reported CVID_QoL score similar to the other groups.

A better self-reported HRQoL has been described among patients who switched from IVIG to SCIG self-infusions at home [33–35], even though this improvement seems to be largely related to a switch to home-therapy rather to the SCIG therapy itself [36–38]. Moreover, several studies evaluating health economics in SCIG and IVIG therapy reported that SCIG was a more cost-effective modality, mainly through the reduction of productivity loss and days in hospital [33, 39–42]. On the other hand, in previous studies analyzing HRQoL after shifting from IVIG to SCIG, it was demonstrated that elderly patients’ approach to SCIG home therapy could be limited by several factors, including the perception of inconvenience of time consumed with self-administration, the aversion to undertake subcutaneous self-injection, or by the anxiety of possible adverse reactions at home [43]. This was confirmed also by our study where older patients still preferred the IVIG treatment.

A limitation of our study is that, at present, the CVID_QoL questionnaire has only been validated in an Italian context. An US English version has been also provided by the Texas Children Hospital group [10]. Study on translation and cross-cultural validation is currently ongoing in several languages, including UK and US English, Deutsch, Dutch, Spanish, Portuguese, Persian, Norwegian, and Greek. A second limitation is the lack of longitudinal data on HRQoL of patients who changed their IgRT.

Independently from the IgRT schedule of administration, the psychological status of participants was in general highly compromised, with very high percentages of positive cases by the GHQ-12 assessment. Moreover, the GHQ-12 positive status has been here identified as a major factor impacting on answers to treatment-related CVID QoL items. This confirmed our previous data on a link between GHQ-12 and HRQoL deteriorations [8, 9]. These data further stress the necessity to monitor the psychological status in CVID, in order to undertake measures to afford anxiety and depression. The strong impact of psychological status on the burden of treatment underlines the need to include instruments addressing the psychological status of patients before drawing any conclusion on the impact of IgRT on HRQoL.

## Electronic Supplementary Material

ESM 1 (DOCX 18 kb)

## Abbreviations

HRQoL: Health-related quality of life
IgRT: Immunoglobulin replacement therapy
GHQ-12: General Health Questionnaire-12
CVID: Common variable immune deficiencies
IVIG: Intravenous immunoglobulins
SCIG: Subcutaneous immunoglobulins
fSCIG: facilitated subcutaneous immunoglobulins
IgG TL: Immunoglobulin G trough levels
CVID_QoL: Common Variable Immune Deficiencies Quality of Life questionnaire
EF: Emotional functioning
RF: Relational functioning
GSS: Gastrointestinal and skin symptoms
COPD: Chronic obstructive pulmonary disease
