# Supplementary material for: Health-Related Quality of Life in Patients with CVID Under Different Schedules of Immunoglobulin Administration: Prospective Multicenter Study
Source: J Clin Immunol. 2019 Jan 15;39(2):159–70. doi: 10.1007/s10875-019-0592-5 (PMC6445807; doi:10.1007/s10875-019-0592-5)
Supplement: Supplementary file 1 — (DOCX 18 kb) [file 10875_2019_592_MOESM1_ESM.docx]

**Supplementary materials**

**Supplementary Table 1.** Content of CVID_QoL questionnaire by dimensions.

| **Emotional Functioning** | |
| --- | --- |
| 1 | Sad |
| 3 | Anger |
| 5 | Difficulty Planning |
| 8 | Health Exacerbation |
| 9 | Joint Pain |
| 10 | Needing Help |
| 12 | Afraid of Adverse Reaction |
| 13 | Concerned About Future |
| 15 | Loss of Autonomy |
| 17 | Difficulty in Usual Activities |
| 18 | Fear of Death |
| 21 | Fear of illness |
| 22 | Weakness |
| 24 | Bothered by Immunoglobulins |
| 28 | Perception as Sick |
| 29 | Embarrassed |
| 30 | Becoming Infected |
| 31 | Troubled by Other Patients |
| 32 | Tired |
| **Relational Functioning** | |
| 6 | Cough |
| 7 | Unable to Provide care |
| 11 | Run Out of Medications/Immunoglobulins |
| 16 | As Contagious |
| 19 | Limited by Cough |
| 20 | Isolated |
| 23 | Difficulty in Sexual Relation |
| 25 | Limitation Upon Leisure Activity |
| 27 | Difficulty in Relationships |
| **Gastrointestinal Skin Symptoms** | |
| 2 | Dietary Changes |
| 4 | Diarrhea |
| 14 | Limited by Diarrhea |
| 26 | Skin diseases |

**Supplementary Table 2.** Reason for changing the Ig route of administration established at T0 during the observational period.

| **Ig route of administration established at T0** | SCIG | IVIG | fSCIG | SCIG+ IVIG |
| --- | --- | --- | --- | --- |
| Refuse to assume responsibility for his/her own health | 2 | 0 | 1 | 0 |
| Fear of not having contact with care giver | 1 | 0 | 3 | 0 |
| Refuse to self-administer | 1 | 0 | 2 | 0 |
| Apprehension on side effects away from medical assistance | 3 | - | 4 | 0 |
| Low compliance with treatment. medical assessment | 2 | 0 | 0 | 0 |
| Side effect (medical assessment) | 2 | 0 | 1 | 0 |
| Lack of family support | 1 | 0 | 0 | 0 |
| Inconvenient treatment | 5 | 2 | 1 | 0 |
| Refusal to continue Ig replacement | 0 | 2 | 0 | 0 |

**Supplementary Table 3.** Characteristics and HRQoL of CVID patients receiving IVIG at T2. Patient groups were selected according to the concentration of intravenous human immunoglobulin (5% and 10%) they were receiving at the study time.

|  | **IVIG concentration** | | **P value** |
| --- | --- | --- | --- |
|  | **10 (g/dL)** | **5 (g/dL)** |  |
| **Age,** year; mean (SD) | 48.0 (15.3) | 51.4 (15.2) | 0.314 |
| **Sex** (females); n (%) | 13 (50.0) | 55 (55.0) | 0.945 |
| **Cumulative monthly Ig dose** (mg/kg); mean (SD) | 336.8 (167.3) | 339.1 (136.8) | 0.943 |
| **Number of monthly administrations**; mean (SD) | 1.9 (0.7) | 2.0 (0.9) | 0.894 |
| **Patients receiving antibiotic prophylaxis**; n (%) | 5 (19.2) | 0 (0) | 0.948 |
| **Time (years) from CVID diagnosis**; mean (SD) | 12.5 (11.3) | 14.9 (11.1) | 0.337 |
| **IgG trough serum levels** (mg/dL); mean (SD) | 670.0 (191.1) | 672.4 (148.4) | 0.946 |
|  |  |  |  |
| **Diarrhea**. episodes-year; mean (SD) | 3.9 (2.9) | 3.8 (2.9) | 0.907 |
| **Sinusitis.** episodes-year; mean (SD) | 1.7 (1.8) | 1.9 (1.7) | 0.671 |
| **Bronchitis.** episodes-year; mean (SD) | 1.5 (1.6) | 1.7 (1.6) | 0.530 |
| **Otitis**. episodes-year; mean (SD) | 0.7 (1.5) | 0.5 (1.0) | 0.409 |
| **Pneumoniae.** episodes-year; mean (SD) | 0.4 (0.8) | 0.2 (0.5) | 0.101 |
| **All infections.** episodes-year; mean (SD) | 5.3 (5.2) | 4.9 (4.8) | 0.718 |
| **COPD**; n (%) | 30 (30.0) | 16 (61.5) | 0.405 |
| **CVID-complication**, cumulative number; mean (SD) | 2.3 (1.4) | 2.2 (1.6) | 0.842 |
|  |  |  |  |
| **GHQ score**; mean (SD) | 14.1 (8.5) | 12.7 (6.4) | 0.413 |
| **CVID_QOL** (%); mean (SD) | 27 (15) | 29 (18) | 0.635 |
| **EF** (%); mean (SD) | 31 (17) | 31 (19) | 0.968 |
| **GSS** (%); mean (SD) | 24 (16) | 28 (20) | 0.418 |
| **RF** (%); mean (SD) | 21 (15) | 25 (18) | 0.296 |

Abbreviation: COPD Chronic Obstructive Pulmonary Diseases, CVID Common Variable Immunodeficiency. CVID_QoL Common Variable Immunodeficiency Quality of Life questionnaire. EF Emotional Functioning. IVIG Intravenously-administered immunoglobulins. GHQ-12 GHQ-12 questionnaire. GSS Gastrointestinal and skin Symptoms. IgRT immunoglobulin replacement treatment, RF Relational Functioning, SD Standard Deviation.
